# 

**Published:** 1938

**Authors:** 


### Bristol Medico-Chirurgical Society

13th April.

Dr. T. M. Ling: "**The Emotional Factor in Disease.**" (Page 101.)

11th May.

Mr. F. W. Willway: "**Problems in the Diagnosis and Treatment of Intracranial Lesions.**"

### The Medical Library of the University of Bristol

WITH WHICH IS INCORPORATED

**The Library of the Bristol Medico-Chirurgical Society.**

*The following donations have been received since the publication of the list in April,* 1938.

July, 1938.

| | | |
|---|---|---|
| Professor E. W. Hey Groves (1) | .. .. | 4 volumes. |
| Mrs. M. Homfray (2) | .. .. .. | 1 volume. |
| Liverpool School of Tropical Medicine (3) | .. | 1 ,, |

Unbound periodicals have also been received from Professor E. W. Hey Groves, Dr. T. Milling, Professor C. Bruce Perry and Dr. J. Odery Symes.

### THE ONE HUNDRED AND SIXTY-THIRD LIST OF BOOKS.

The figures in round brackets refer to the figures after the names of the donors. The books to which no such figures have been attached have either been bought from the Library Fund or received through the *Journal.*

**Antill, J. M. and Warren, R. A.** *The Emancipist* .. .. (1) 1936

**Appleton, A. B., and others.** *Surface and Radiological Anatomy* .. 1938

**Bailey, Hamilton** .. *Emergency Surgery* .. .. 3rd Ed. (1) 1938

**Brasher, C. W. J.** .. *Faith Healing* .. .. .. .. .. 1938

**Buchanan, Scott** .. *The Doctrine of Signatures* .. .. .. 1938

**Byrne, J. Grandson** .. *Studies on the Physiology of the Middle Ear*.. 1938

**Cope, Z.** .. .. .. .. *Actinomycosis* .. .. .. .. .. 1938

**Geckeler, E. O.** .. *Fractures and Dislocations for Practitioners* (1) 1937

**Gray, H.** .. .. .. .. *Anatomy, Descriptive and Surgical* .. (2) 1858

**Hopewell-Ash, E. L.** *Manipulative Methods in the Treatment of Functional Disease* .. .. .. .. 1935

**James, P. R.** .. .. *The Baths of Bath* .. .. .. .. 1938

**Mathews, A. P.** .. *Principles of Biochemistry* .. .. .. 1936

*Modern Treatment in General Practice,* Vol. IV .. .. .. 1938

*Myocarditis—the St. Cyres Memorial Lectures* .. .. .. .. 1937

**Nordmann, O.** .. .. *Praktikum der Chirurgie* .. 4th Ed. (1) 1938

**V. Norman**.. .. .. *Essentials of Modern Medical Treatment* .. 1936

*Occupational Therapy—An Addendum to the Handbook for Mental Nurses* 1938

**P. E. P.** .. .. .. *Report on the British Health Services* .. 1937

**Philip, Sir R.** .. .. *Collected Papers on Tuberculosis* .. .. 1937

**Rea, R. Lindsay** .. *Neuro-Ophthalmology* .. .. .. .. 1938

**Rolleston, Sir H., and Moncrieff, A.A.** *Practical Procedures*.. .. 1938

**Sechehaye, A.** .. .. *The Treatment of Tuberculous Affections with Umckaloabo* .. .. .. .. .. 1938

**Shanks, S. C., and others** (eds.). *Textbook of X-ray Diagnosis by British Authors,* Vols. I-II.. .. .. 1938

**Sleeping Sickness Committee.** *Minutes of Evidence taken by the Departmental Committee on Sleeping Sickness* (3) 1914

*Survey of Chronic Rheumatic Diseases* .. .. .. .. .. 1938

*Treasury of Human Inheritance,* Vol. II, Parts 1–3 .. .. 1922–28

### TRANSACTIONS, REPORTS, JOURNALS, ETC.

Collected papers of the Mayo Clinic, XXIX. (1937) .. .. 1938

Medical Annual, 1938 .. .. .. .. .. .. .. 1938

Quarterly Cumulative Index Medicus, Vol. 22 (1937) .. .. 1938

Transactions of the Ophthalmological Society of the United Kingdom, Vol. LVII., Part 2 .. .. .. .. .. .. .. 1938

### Publications Received

From Messrs. Cassell & Co. Ltd.:

*Sick Children: Diagnosis and Treatment.* By Donald Paterson, B.A. Manitoba, M.D. Edin., F.R.C.P. Lond. 3rd Edition. Price 12s. 6d.

From Messrs. Gerald Duckworth & Co. Ltd.:

*Faith Healing.* By C. W. J. Brasher, M.D. Price 5s.

From Messrs. B. Fraser & Co.

*The Treatment of Tuberculous Affections with Umckaloabo.* By Adrien Sechehaye. Price 1s.

From Messrs. Hodder & Stoughton, Ltd.:

*Introduction to Diseases of the Chest.* By James Maxwell, M.D., F.R.C.P. Price 12s. 6d.

From Keesing's Medical Digest, Ltd.:

*Keesing's Medical Digest.* January 1st to March 31st, 1938. Annual price £1 1s.

From Messrs. Kegan Paul, Trench, Trubner & Co. Ltd.:

*The Doctrine of Signatures.* By Scott Buchanan. Price 7s. 6d.

From Messrs. H. K. Lewis & Co. Ltd.:

*The Evolution of Chronic Rheumatism.* By R. Fortescue Fox, M.D. (Lond.), F.R.C.P. Price 2s. 6d.

From Dr. M. F. Liston:

*Non-Venereal Syphilis.* By M. F. Liston, O.B.E., M.B., Ch.B.

From Messrs. E. and S. Livingstone:

*Medicine for Nurses.* By C. Bruce Perry, M.D., F.R.C.P. Price 5s.

From Oxford University Press:

*Heart Disease and Pregnancy.* By Crighton Bramwell, M.A., M.D., F.R.C.P., and Edith A. Longson, M.B., Ch.B., D.P.H. Price 8s. 6d.

*The History of the Forceps.* By Vilhelm Møller-Christensen. Price 20s.

*Textbook of Nutrition.* By J. A. Nixon, C.M.G., M.D., F.R.C.P., and Doreen G. C. Nixon, M.B., B.S., M.R.C.S., L.R.C.P. Price 7s. 6d.

From Messrs. John Wright & Sons, Ltd.:

*British Journal of Surgery,* Vol. XXV., No. 100. April, 1938. Price 12s. 6d.

*A Synopsis of Physiology.* 3rd Edition. By A. Rendle Short, B.Sc., M.D., F.R.C.S., and C. L. G. Pratt, M.A., M.Sc., M.D. Price 10s. 6d.

*Anæsthesia and Analgesia for Nurses and Midwives.* By J. K. Watson, M.D. Price 3s. 6d.

### Local Medical Notes

EXAMINATION RESULTS.

**University of Bristol.**—Students of the University have recently passed the following examinations :—

M.D.—Mabel F. Potter.

M.B., Ch.B.—*Second Examination. Pass :* G. S. Andrews (with distinction in Anatomy), J. L. Ashley, Marie L. Bartlett (with distinction in Physiology and Anatomy), R. C. Blackney, J. Brodie, Joyce W. Brown, K. L. Brown, N. J. Brown, J. D. Cardale, J. H. P. Davies-Sage (with distinction in Anatomy), Cecil M. Drillien, D. I. Fullerton, S. J. V. Gilson, F. J. Goddard, Hester M. Hammond, Kathleen M. A. Harland, Joyce Kennard, P. Legat, D. S. Maunsell, A. H. M. Oakes, Christine M. Rendell, F. B. Richards (with distinction in Anatomy), A. F. Rogers, A. R. Rowe, M. O. Skelton, J. C. Valentine, G. A. Walton, J. H. Wilkins.

M.B., Ch.B., Section I.—*Final Examination. Pass :* Ruth M. Hunter.

M.B., Ch.B., Section II.—*Final Examination. Pass :* Kathleen E. Byrt (with distinction in Forensic Medicine and Toxicology), K. J. V. Carlson, Margaret H. Davies, Eileen M. D. Knox, G. R. E. Maxted, Margaret E. Morgan, R. H. Owen (with distinction in Forensic Medicine and Toxicology), Mabel W. N. Tribe, A. H. Wright, W. H. G. Elliott, J. S. Richardson.

B.D.S.—*Second Examination. Pass :* F. C. Bryant, P. B. Carey, Margaret J. Griffith, G. K. G. Jennings (with distinction in Anatomy), Sarah Jones, P. M. Nicholas (with distinction in Anatomy), R. M. Sharp.

B.D.S.—*Third Examination. Pass :* F. C. Baker.

B.D.S.—*Final Examination. Pass :* F. P. Ewens.

L.D.S.—*Second Examination. Pass :* Jean M. Bryant, R. L. Hollington, G. J. Mills, V. T. Scard.

L.D.S.—*Third Examination. Pass :* D. G. Davies, P. Le Masurier, J. W. S. Jenkins, L. H. Barker-Tufft, J. R. Herbert, L. B. Howell, O. H. Minton, C. R. Sage, J. C. Jowett, R. B. Stiles.

L.D.S.—*Final Examination. Pass :* F. S. S. Baguley, J. F. Durie, C. F. Howell, C. C. Moore, L. K. A. S. Oxley, D. M. Sanders, R. V. Woods.

MIDWIFE TEACHERS' CERTIFICATE EXAMINATION.—PART II. *Pass :* Lilian M. Phillips.

**Conjoint Board.**—L.R.C.P., M.R.C.S.—*Pass :* J. L. Reynolds (Anatomy), Edith M. Wagstaff (Pharmacology and Materia Medica), Joyce S. Miller (Pharmacology and Materia Medica), *Ursula W. Wood (Medicine), *Myrtle M. Hutchins (Medicine), R. T. Moore (Pathology and Midwifery), Margaret E. M. Slater (Surgery).

* Qualified.

---

APPOINTMENTS.

Miss Helen Bell, Matron at Northampton General Hospital, has been appointed Matron at the Bristol Royal Infirmary.

Miss A. Cameron, Matron at Hereford Hospital, has been appointed Matron at St. Monica's Home of Rest.

